# Improved metabolic function and cognitive performance in middle-aged adults following a single dose of wild blueberry

**DOI:** 10.1007/s00394-020-02336-8

**Published:** 2020-08-03

**Authors:** Adrian R. Whyte, Sajida Rahman, Lynne Bell, Indika Edirisinghe, Robert Krikorian, Claire M. Williams, Britt Burton-Freeman

**Affiliations:** 1 School of Psychology and Clinical Language Sciences, University of Reading, Earley Gate, Whiteknights, Reading, RG6 6AL UK; 2 Centre for Nutrition Research, Department of Food Science and Nutrition, Illinois Institute of Technology, Chicago, IL USA; 3 Department of Psychiatry and Behavioral Neuroscience, University of Cincinnati Academic Health Centre, Cincinnati, OH USA

**Keywords:** Wild blueberry, Cognition, Executive function, Glucose, Insulin, Fruit, Polyphenols

## Abstract

**Purpose:**

Research has demonstrated cognitive benefits following acute polyphenol-rich berry consumption in children and young adults. Berry intake also has been associated with metabolic benefits. No study has yet examined cognitive performance in middle-aged adults. We investigated the relationships among cognitive and metabolic outcomes in middle-aged adults following wild blueberry (WBB) consumption.

**Methods:**

Thirty-five individuals aged 40–65 years participated in a randomized, double blind, cross-over study. Participants consumed a breakfast meal and 1-cup equivalent WBB drink or matched placebo beverage on two occasions. Participants completed cognitive tasks and had blood drawn before and at regular intervals for 8 h after each meal/treatment. Changes in episodic memory and executive function (EF) were assessed alongside plasma levels of glucose, insulin, and triglyceride.

**Results:**

Analysis of the memory-related Auditory Verbal Learning Task (AVLT) word recognition measure revealed a decrease in performance over the test day after placebo intake, whereas performance after WBB was maintained. For the AVLT word rejection measure, participants identified more foils following WBB in comparison to placebo. Benefits were also observed for EF on the Go/No-Go task with fewer errors following WBB intake on cognitively demanding invalid No-Go trials in comparison to placebo. Furthermore, in comparison to placebo, response times were faster for the Go/No-Go task, specifically at 4 h and 8 h following WBB treatment. We also observed reduced post-meal glucose and insulin, but not triglyceride, concentrations in comparison to placebo over the first 2 h following ingestion. Though the addition of Age, BMI, glucose and insulin as covariates to the analysis reduced the significant effect of beverage for AVLT word rejection, metabolic outcomes did not interact with treatment to predict cognitive performance with the exception of one isolated trend.

**Conclusions:**

This study indicated acute cognitive benefits of WBB intake in cognitively healthy middle-aged individuals, particularly in the context of demanding tasks and cognitive fatigue. WBB improved glucose and insulin responses to a meal. Further research is required to elucidate the underlying mechanism by which WBB improves cognitive function.

## Introduction

There is a substantial body of evidence demonstrating an association between habitual consumption of foods high in flavonoids and cognitive benefits [1] including delayed cognitive decline with ageing [2, 3]. In addition, evidence from controlled intervention trials corroborate these findings, showing that supplementation with flavonoid-rich foods produce improvements in cognitive performance (for reviews see [4, 5]).

The majority of human berry trials have investigated the effects of supplementation for periods of several weeks [6–12], although recent data suggest that effects of other flavonoid-rich food, such as cocoa, on brain function [13, 14] and neurocognitive function [15, 16] can occur within hours of consumption. Likewise, in school-aged children, whole fruit blueberry powder delivered in a smoothie/juice drink was associated with improvements in executive function and memory performance 2–6 h following intake [17–20]. In young adults, a smoothie of equal blueberry, strawberry, raspberry, and blackberry, was associated with improvements in executive function, again 2–6 h following intake [21]. Furthermore, in older adults, global cognitive function was found to decline relative to baseline at 2 h following a control beverage whereas performance was maintained for a flavonoid-rich blueberry beverage [22]. Importantly, these improvements in cognitive performance were demonstrated following berry intake at intervals similar to plasma peaks of blueberry anthocyanins and their metabolites 1–3 h after ingestion, as well as plasma peaks of different phenolic acid metabolites 2–3 h and 5 h post-consumption [23–25].

Related to these timescales, reduced postprandial insulin (1–3 h) and attenuated postprandial inflammation have been shown to occur for up to 10 h in middle-aged, overweight and obese individuals consuming strawberries with a typical Western meal, and in a younger overweight group consuming strawberries 2 h before the meal, respectively [26, 27]. Similarly, red raspberry intake with a high carbohydrate breakfast meal reduced postprandial glycaemia and the concomitant insulin demand in overweight or obese individuals with pre-diabetes and insulin resistance [28] across time frames that anthocyanin and phenolic metabolites were apparent in blood [29]. Further, in a dose response study in individuals with obesity and insulin resistance, insulin and glucose responses after strawberry intake with a meal were associated with the main anthocyanin metabolite of strawberry, pelargonidin glucuronide [30].

Collectively, there is evidence suggesting that cognitive benefits after a single administration of polyphenol-containing berry fruits occurs during a timeframe corresponding to both the pharmacokinetic profiles of berry (poly)phenols and biological effects associated with metabolic health. Notably, observational data suggest a strong link between metabolic syndrome and cognitive impairment [31], suggesting that dietary components and/or their metabolites that impact metabolic systems, also might impact cognitive function. However, these factors and their relationships have not been investigated in a clinical trial. Also, while evidence of immediate cognitive benefit following polyphenol intake has been observed in school-aged children, young adults, and older adults, there is limited research to date concerning such cognitive effects in middle-age, a period that is noteworthy because of the association of mid-life health conditions, particularly metabolic disturbance, with risk for late-life dementia [32]. Therefore, the aim of this study was to examine relationships among cognitive performance and metabolic responses in middle-age adults following one-time intake of whole fruit wild blueberry powder.

## Method

The study was registered with identifier NCT02736331 on ClinicalTrials.gov, and participants reviewed and signed an informed consent document approved by the Institutional Review Board, Illinois Institute of Technology, Chicago, IL, USA. The study was performed in accordance with the ethical standards laid down in the 1964 Declaration of Helsinki and its later amendments. Study participants were recruited from the Greater Chicagoland area, and study visits were conducted at the Clinical Nutrition Research Centre (CNRC) at Illinois Institute of Technology, Chicago, IL, USA.

### Participants

We advertised for participants who were middle aged and who were between 40 and 65 years old with BMI between 18.5 and 34.9 kg/m^2^. Participants were required to be non-smokers for at least two years, able to understand the requirements of the cognitive function tasks, not taking medications that might interfere with study outcomes such as glycaemic, lipid-lowering, and psychostimulant medications, and without cardiovascular, respiratory, renal, gastrointestinal, and neurological disorders.

Sixty-five individuals were screened for the study and 25 were disqualified for the following reasons, ineligible BMI (*n* = 1), medical concerns (*n* = 7), failure to adequately perform cognitive tasks (*n* = 1), recent/current smoker (*n* = 3), lifestyle patterns that would influence study objectives (*n* = 3), loss to follow up (*n* = 3), and withdrawal of consent (*n* = 7). Of the 40 participants who were randomized, 3 participants were withdrawn due to medical concerns and another 2 due to poor adherence to study procedures.

### Study design and experimental beverages

The study was a randomized investigator- and subject-blinded, 2-arm, placebo-controlled, cross-over trial. Participants received one of the experimental beverages with a breakfast meal on two occasions separated by at least **7** days determined by a computer generated randomization sequence. The study visits required 9–9.5 h subjects’ time and followed an identical protocol with the exception of the administered drink: wild blueberry or placebo beverages.

The wild blueberry beverage consisted of 25 g freeze-dried whole wild blueberry (WBB) powder (~ 1-cup fresh weight) sourced from the Wild Blueberry Association of North America, Old Town, Maine, USA and freeze-dried, analysed and packaged by FutureCeuticals, Momence, IL. The WBB powder was mixed with water, frozen lemonade concentrate, and unsweetened Kool-aid for colour and flavouring. The placebo beverage was matched as closely as possible for energy and macronutrient content using the same ingredients minus the WBB powder. The breakfast meal included buttermilk biscuits with unsalted butter and apple jelly, scrambled eggs, and honeydew melon balls. The breakfast meal and beverages were consumed together. Table 1 contains the nutritional composition of the beverages and Table 2 provides the nutritional composition of the breakfast meal.

**Table 1:** Nutritional composition of treatment beverages^a^

Nutrient	WBB beverage^b^	Control beverage^c^
Energy (kcal)	153	152
Carb (g)	33	34
Protein (g)	1	0
Fat (g)	1	0
Fibre (g)	4	0
Sugar (g)	28	32
Glucose^d^ Fructose^d^	14 14	16 16
Polyphenols (mg)^e^	725	0
Anthocyanins (mg)^e^	475	0
Energy density (kcal/g)	0.4	0.4

^a^Beverages were served with the standardized breakfast. Data are derived from ESHA software, Salem, Oregon, USA methods section

^b^WBB Beverage (348 g): freeze-dried WBB powder, lemonade frozen fruit concentrate (Minute Maid, Sugarland, TX), Black Cherry Kool-aid drink mix (Kraft, Northfield IL), Grape Kool-aid drink mix (Kraft, Northfield, IL) and water

^c^Control Beverage (348 g): lemonade frozen fruit concentrate (Minute Maid, Sugarland, TX), Black Cherry Kool-aid drink mix (Kraft, Northfield IL), Grape Kool-aid drink mix (Kraft, Northfield, IL) and water

^d^Values are provided by Reading Scientific Services LTD, Reading UK

^e^Values are calculated from certificate of analysis provided by Futureceuticals, Momence, Indiana: total polyphenols present in powder at 2.9% by FCCM C.2.4 method and total anthocyanins present in powder at 1.9% by FCCM C.13.1 method

**Table 2:** Nutritional composition of standardized meals^a^

Nutrient	Standardized breakfast^b^	Standardized light lunch^c^
Energy (kcal)	690	249
Carb (g)	88	11
Protein (g)	14	8
Fat (g)	33	20
Fibre (g)	1	3
Sugar (g)	44	5
Polyphenols (mg)	< 1	< 1
Anthocyanins (mg)	0	0
Energy density (kcal/g)	1.8	1.0

^a^Standardized breakfast was served with treatment beverages. All meals analysed by ESHA software 2018, Version 11.6.522, Salem, Oregon, USAand PhenolExplorer for polyphenols content

^b^Breakfast meal included buttermilk biscuits (Pillsbury General Mills, Minneapolis, MN), unsalted butter (Ahold USA INC, Landover, MD), apple jelly (Smuckers, Orrville, OH), scrambled eggs (Dutch Farms, Chicago, IL), and fresh honeydew melon balls

^c^Light Lunch was served after the 6 h blood collection and cognitive assessment. Lunch included freshly sliced cucumbers without skin, Cucumber Ranch salad dressing (Kraft, Northfield, IL), fresh parsley, and Lightly Salted Cocktail Peanuts (Planters, Wilkes-Barre, PA)

### Study procedures

After screening and enrolment, all participants underwent a pre-study visit to obtain 3-day dietary records and become familiar with study day cognitive tasks. Dietary records were collected to counsel participants on convenient ways to comply with avoiding berry products and high polyphenol/anthocyanin foods during the study. To minimize practice effects participants were administered two full practice versions with the cognitive tasks during the pre-study visit.

After the pre-study visit, participants were scheduled for the first study visit. Study procedures required participants to maintain usual dietary and activity habits throughout the experimental period, except that berry products were to be avoided for at least seven days prior to each study visit. Preparation the day before each study required participants to consume 8–10 cups of water and avoid alcohol 24-h before each study day. The evening meal before a study day visit was controlled by limiting participants’ choice to purchasing a meal from a local sandwich shop or taking a standardized meal from the CNRC. Participants ate the same meal before each study visit. Participants were instructed to begin a water only fasting protocol after 10:00 pm.

**Fig. 1:**
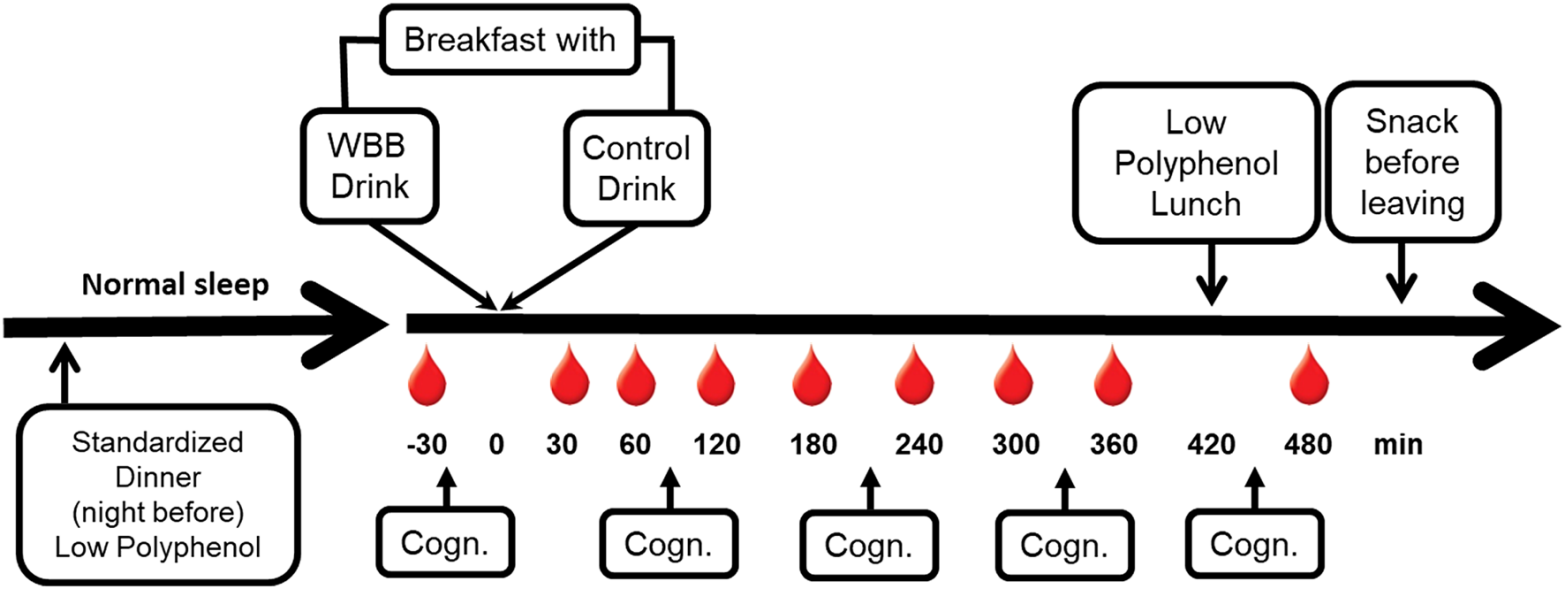
Time line of procedure, blood draws and cognitive testing on test days

Upon arrival at the CNRC for each study day visit, participants were asked to confirm the period of their fast (time documented since last food or drink), and compliance with avoidance of berry and polyphenol/anthocyanin rich foods. Height, body weight, waist circumference, and body temperature were measured. Fasting blood glucose levels were obtained via finger prick, and blood pressure and heart rate were measured three times at five minutes intervals. An intravenous catheter was placed in the non-dominant arm and participants completed the baseline set of cognitive tasks while fasted. Following completion of the baseline assessment, a blood sample was collected and participants were provided the standardized breakfast including either the wild blueberry or placebo beverage based on a randomized sequence. As shown in Fig. 1, blood samples were collected at 30, 60, 120, 180, 240, 300, 360, and 480 min after the start of the meal, and matched versions of the cognitive tasks were repeated at 120, 240, 360, and 480 min after the meal and immediately after blood collection. A lunch consisting of cucumber salad with peanuts was provided at 420 min. All study foods were prepared in the Metabolic Kitchen of CNRC following food safety standards as dictated in the CNRC Standard Operating Procedures. After the 480-min assessments, the catheter was removed and participants were evaluated for safety before discharge.

### Study assessments

#### Cognitive evaluation

E-prime V2 (Psychology Software Tools, Inc) was used to display stimuli and record participants’ responses. Auditory stimuli were presented through noise cancelling enclosed headphones. The cognitive protocol was designed to evaluate executive control functions essential to working memory and new learning and retention (long-term memory). These domains are characteristically vulnerable to decline with aging beginning in midlife [33]. The protocol included two tasks measuring executive abilities and one test of episodic memory.

A computer-administered version of the Auditory Verbal Learning Task (AVLT) examined learning, recall and recognition memory [18, 34]. Such a word list learning task generally makes lower demand on executive ability than other types of learning and memory tasks such as complex figure and paragraph recall, tasks that involve integration and organization of complex material [35]. Participants were presented with five auditory exposure and immediate oral recall trials of a pre-recorded list of 15 common words (list A, trials 1–5) followed by one exposure and free recall trial of an interference list of 15 words (list B). After the recall for list B and without further exposure to list A, another recall trial (list A, trial 6) for list A was elicited. Following a 15 min delay interval, during which participants engaged in other cognitive tasks, participants were asked to recall the items from list A again. Immediately following this recall trial, participants performed a word recognition task where they were exposed to a list of 50 words displayed one at a time on the screen containing the words from lists A and B along with 20 foils. They were asked to respond by pressing a green ‘Y’ key when shown words from list A and a red ‘N’ key when shown words from list B or the 20 other foils. Ten balanced word lists were used in this study (as previously reported [18]) with list presentation randomized using a balanced Latin square across the 10 cognitive test sessions.

Measures drawn from the AVLT included: (1) immediate recall following list A trial 1; (2) total number of words learned (a measure of the number of additional words learned between list A trial 1 and list A trial 5, calculated as recall in trial 5 minus recall in trial 1); (3) final acquisition (a measure of the total number of words learned following all 5 presentations of list A, calculated as recall in trial 5); (4) proactive interference (a measure of the effect of previously encoded information on the acquisition of new information calculated as the number of words recalled from list B minus number of words recalled in list A trial 1); (5) retroactive interference (a measure of the effect of newly encoded information on the recall of previously encoded information, calculated as the number of words recalled in list A trial 5 minus number of words recalled in list A trial 6 – note the newly encoded information in this case is the presentation and recall of list B which separates list A, trials 5 and 6); (6) delayed recall (number of list A words recalled after the 15 min delay); (7) word recognition accuracy and reaction time (number of true positives out of the possible 15 list A items); (8) word rejection accuracy and reaction time (number of true negatives out of the possible 35, consisting of the 15 list B items and 20 foils).

The Modified Attention Network Task (MANT) assessed susceptibility to response interference by having participants respond on a keyboard indicating the direction of a stimulus arrow displayed on a computer screen either directly above or below a central fixation cross [19]. Alerting (either a star replacing the fixation cross, or no cue) and orienting (either one star displayed directly above or below the fixation in the position of the next stimulus arrow, or two stars displayed simultaneously directly above and below the fixation cross) cues were presented 120 ms before each stimulus presentation, followed by a 450 ms fixation screen. The stimulus was then presented for 500 ms. Congruence was manipulated by the direction of the central arrow which could either be congruent (same direction) or incongruent (opposite direction) to the surrounding arrows. Visual load was manipulated by the number of flanking arrows which could either be 10, 5 or none. An inter-stimulus interval fixation screen was then displayed for 1000 ms before the next cue. Measures of accuracy and reaction time were gathered by congruency and load.

Finally, the Cued Go/No-Go task [36] examined response inhibition by having participants respond to empty cue rectangles. Following a variable duration of 100, 200, 300, 400 or 500 ms, the cue rectangles were then filled with green “go” or blue “no-go” stimuli. Vertical rectangles were followed on 80% of trials by the blue “no-go” stimuli (valid “no-go” cues), while the remaining 20% of vertical rectangles were followed by green “go” stimuli (invalid “go” cues). Horizontal rectangles were followed on 80% of trials by a green ‘go” stimuli (valid “go” cues), the remaining 20% of horizontal rectangles were followed by blue “no-go” stimuli (invalid “no-go” cues). Thus, the orientation of the rectangle cued the participant to the predominant stimulus type that followed. Where a vertical “no-go” cue rectangle is followed by a go stimulus slower reaction times were expected due to the additional cognitive demand placed on the participant to overcome the pre-potent prime to withhold a response. Conversely where a horizontal “go” cue is followed by a “no-go” stimulus, increased errors of commission were expected due to the additional cognitive demand placed on the participant to overcome the pre-potent prime to make a “go” response. Reaction time by cue type was measured for “go” stimuli and number of commission errors by cue type was measured for “no-go” stimuli.

#### Metabolic and lipid assays

Blood samples were collected in vacutainer tubes coated with ethylenediaminetetraacetic acid (EDTA). Samples were centrifuged at 453 × g for 15 min at 4  °C. Plasma was collected and stored at  – 80 °C until analysed. Glucose, insulin, and triglyceride levels were measured using enzymatic calorimetric methods (Randox, UK – Cat # 3815), immunoturbidimetry assay methods (Kamiya Biomedicals, WA, USA – Cat # KAI-071) and standardized enzyme-based assay methods (Randox, UK – Cat # TR3823), respectively. All assays were performed on the Randox Daytona automated clinical analyser (Randox, UK) according to manufacturer’s instructions. Quality controls were applied throughout sample analysis.

### Statistical analysis

The participant sample characteristics were tabulated using descriptive statistics. Statistical analyses of the cognitive measures were performed using SPSS v25 linear mixed modelling (LMM) employing an unstructured covariance matrix to model successive repeat measurements. ‘Participant’ was included as a random factor to control for non-independence of data within participants. In our primary analysis of the cognitive data, for all cognitive tasks, Visit Order (Visit), Time after experimental beverage (Time), experimental beverage (Beverage), and Time × Beverage interaction were included as fixed factors in the model. For the Cued Go/No-Go, the following additional fixed factors were included; Cue type, Beverage × Cue type, and Beverage × Cue Type × Time. For the MANT the following additional fixed factors were included; Congruency, Load, Beverage × Congruency, Beverage × Load, Beverage × Congruency × Load, Beverage × Congruency × Time, Beverage × Load × Time, and Beverage × Congruency × Load × Time.

Metabolic and lipid data were tested for normality using Shapiro–Wilk tests and log transformed when indicated (i.e. insulin, triglyceride). Glucose, insulin and triglyceride measures were analysed by repeated measures analysis of variance (ANOVA) in a mixed model procedure using SAS v9.4. The primary analysis was similar to that performed on cognitive data using Time, Beverage, and Time x Beverage interaction, and Visit, Beverage, and Visit x Beverage interaction as fixed variables and participants as the random factor. Area under the curve analysis (AUC) was performed using total area under the response curve for 480 min as well as in increments of 120 min.

To understand the relationship of metabolic and lipid factors with cognitive outcomes, a secondary LMM analysis included the fixed factors as described above with Insulin, Glucose, BMI, and Age as covariates along with the interaction between each of these covariates and Beverage. AUC for the first 120 min for Insulin and Glucose was utilized in these models based on the hypothesized impact of the initial post-meal metabolic response on downstream cellular and systemic alterations (increased oxidative stress and inflammatory responses) [26, 27].

To estimate statistical power in this preliminary study, we assumed a medium effect size and alpha probability = 0.05. Given the sample size estimate of 35 participants, power = 0.69. Trends in statistical significance were acknowledged with alpha probability 0.05–0.1. In all analyses, multiple pairwise comparisons with Bonferroni correction for familywise error were applied. This included non-significant interactions; which is statistically appropriate where sufficient correction for type 1 error has been employed [37–39].

## Results

### Subject characteristics

Forty individuals were enrolled of which 35 completed the pre-study visit and both study days. The 35 completers had a mean (± SD) age of 50.9 ± 7.8 and BMI of 26.7 ± 4.1 kg/m2. Table 3 contains a summary of participant characteristics.

**Table 3:**
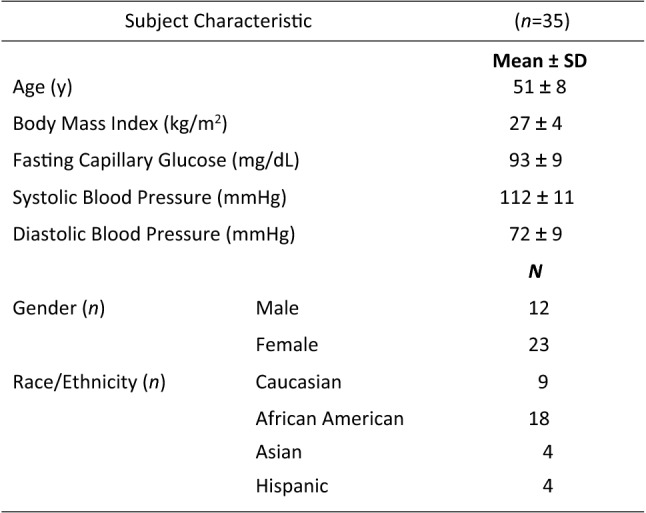
Demographic information of participants

### Cognitive function

#### Auditory verbal learning test

**Fig. 2:**
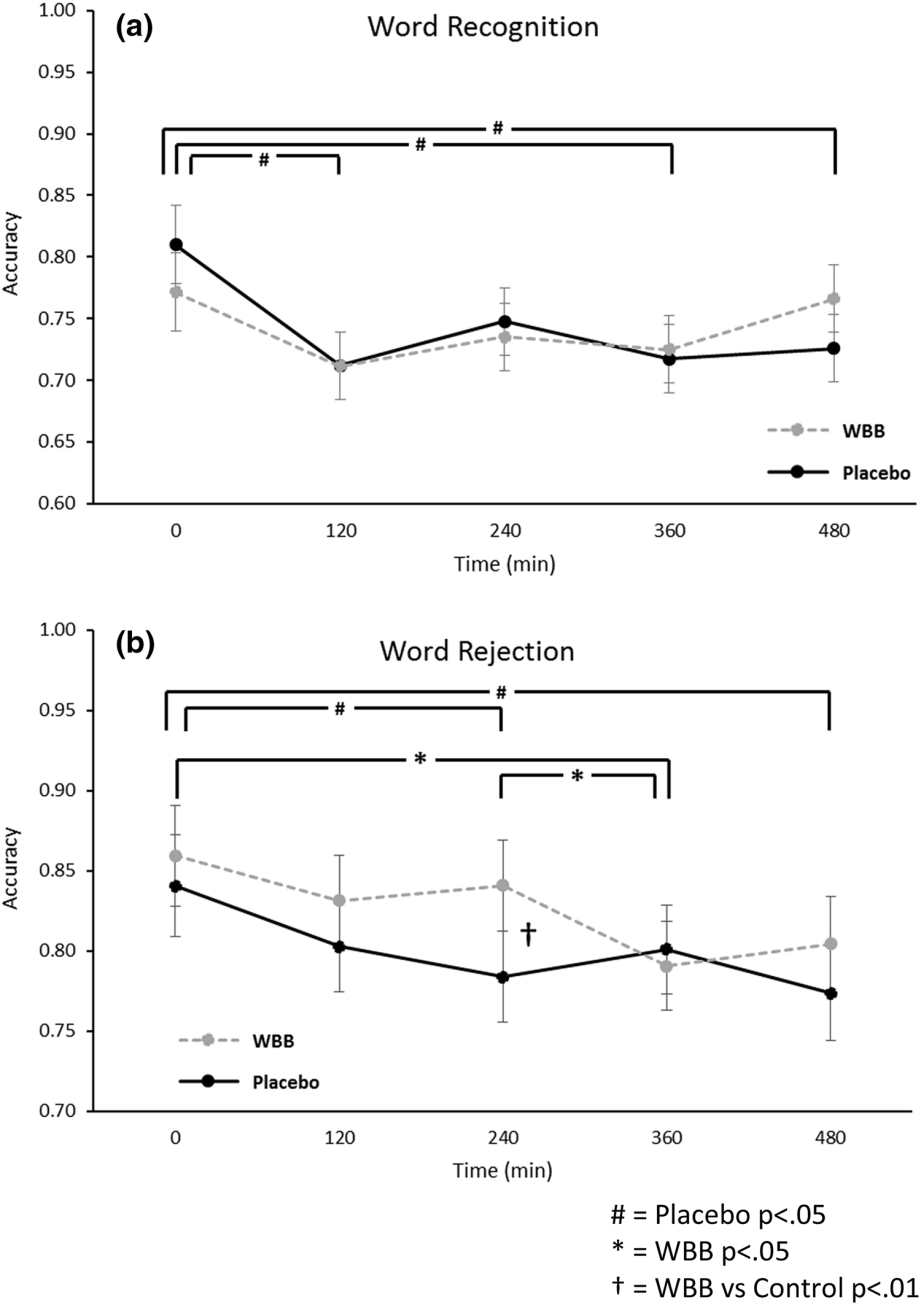
**a** Mean word recognition accuracy (± Standard error of the mean) at all-time points following WBB or control intervention. Placebo accuracy can be seen to significantly decrease between baseline and 120 min, 360 min, and 480 min whereas no such decrease is found for WBB. **b** Mean word rejection accuracy (± Standard error of the mean) at all-time points following WBB or control intervention. Placebo accuracy can be seen to significantly decrease between baseline and 240 min, and baseline and 480 min. WBB accuracy can be seen to significantly decrease between baseline and 360 min and 240 min and 360 min

Significant effects on the AVLT were found only for the recognition memory component of the task where participants were asked to identify from a list of 50 words those that had been presented in the initial word list (from trial 1), the interference word list (list B), and 20 novel foils. As would be expected in relation to cumulative interference effects, both Visit [*F*(1, 34.5) = 8.74, *p* = 0.006], with participants performing less accurately at Visit 2 (*M* = 0.716) in comparison to Visit 1 (*M* = 0.769), and Time [*F*(1, 70) = 3.84, *p* = 0.007], with participants performing less well over the course of the day, predicted accuracy of word recognition. Beverage [*F*(1, 36.3) = 0.002, *p* = 0.97] and the Beverage x Time interaction [*F*(4, 70) = 1.084, *p* = 0.371] failed to reach significance. However, as shown in Fig. 2a, pairwise comparisons revealed a significant reduction in accuracy for the placebo condition between baseline and 120 min [*p* = 0.015], 360 min [*p* = 0.02] and 480 min [*p* = 0.043]. No such reduction in accuracy was found for the WBB condition where recognition performance was not different at any post-ingestion assessment point.

For performance based on accuracy of word rejection, Visit and Time significantly predicted performance [*F*(1, 35.4) = 4.56, *p* = 0.04; *F*(4,70) = 4.96, *p* = 0.001, respectively] with participants performing more accurately at Visit 2 (*M* = 0.822) in comparison to Visit 1 (*M* = 0.804), and with participants performing worse (i.e., less accurately) over the course of the day as shown in Fig. 2b. In addition, there was an effect of Beverage [*F*(1, 39.5) = 6.65, *p* = 0.014] with WBB-treated participants accurately identifying more foil words (*M* = 0.825) than those under placebo conditions (*M* = 0.80). The Beverage x Time interaction trended towards significance [*F*(4, 70) = 2.01, *p* = 0.094], and pairwise comparisons indicated relatively better performance following WBB in comparison to placebo, particularly evident at 240 min [*p* = 0.002]. Significant decreases in accuracy were also found for WBB-treated participants between baseline and 360 min [*p* = 0.02] and 240 min and 360 min [*p* = 0.045], and significant decreases in accuracy for placebo participants between baseline and 240 min [*p* = 0.04] and baseline and 480 min [*p* = 0.033].

There was no effect of the Beverage on measures of learning, proactive interference (PI), retroactive interference (RI), and recognition memory reaction time (RT). Similarly, there was no main effect of Beverage or Beverage × Time interaction on delayed recall, however, pairwise placebo comparisons did show diminished recall associated with both WBB and placebo treatments with the exception of a short-lived improvement following placebo between 120 and 240 min [*p* = 0.047].

#### Modified attention network task

**Fig. 3:**
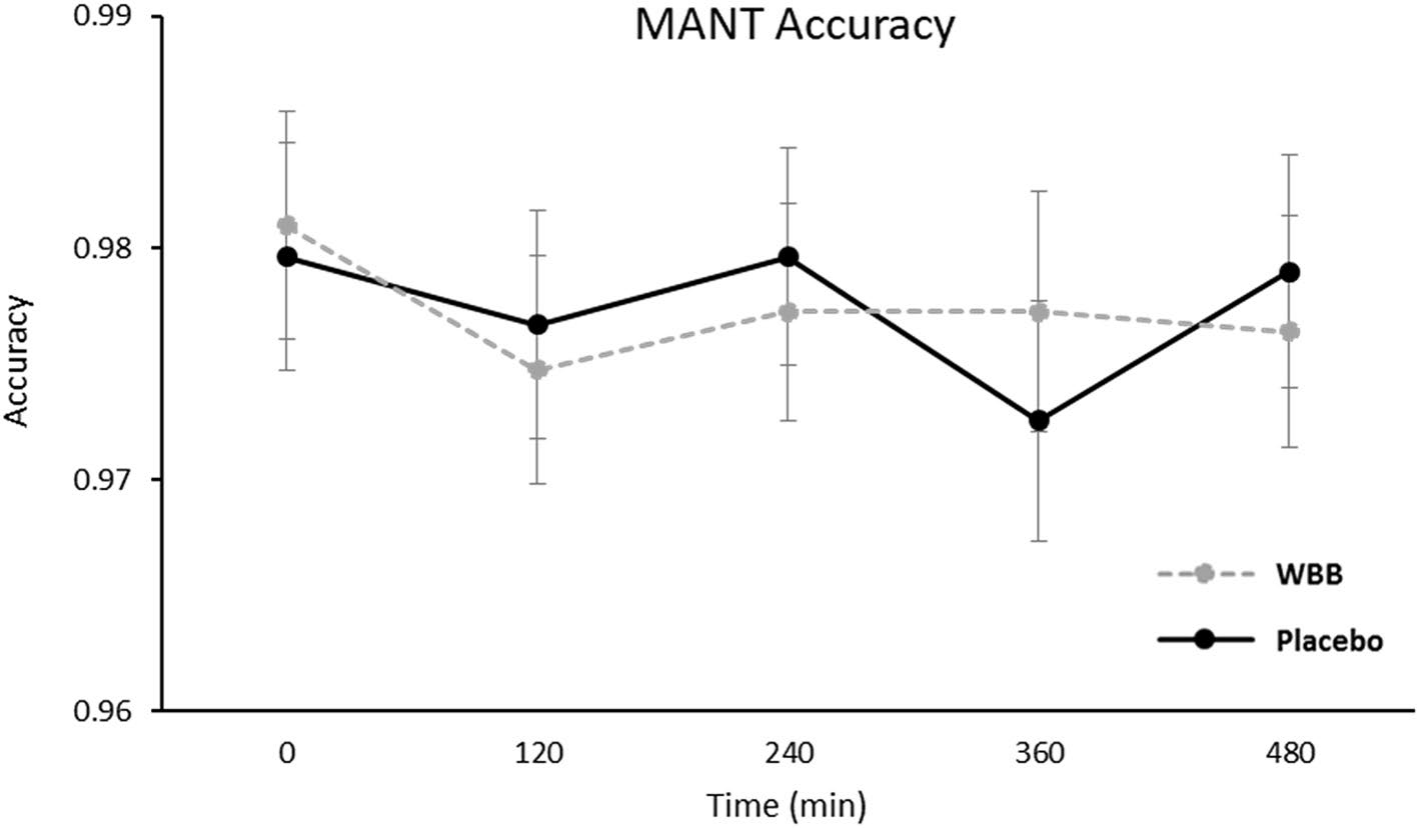
Mean accuracy performance (± Standard error of the mean) at all-time points following WBB or control intervention

As expected, Congruency [*F*(1, 243.7 = 98.4, *p* < 0.001] and Load [*F*(1, 243.7) = 8.22, *p* = 0.004] influenced response accuracy. There was no main effect of Beverage on response accuracy. There was a Beverage × Congruency × Load interaction [*F*(2, 243.68) = 3.16, *p* = 0.044], however, although pairwise comparisons revealed only one significant finding whereby there was a short-lived reduction in accuracy on incongruent, high load trials between 240 and 360 min for the placebo treatment, there were no significant between treatment pairwise comparisons for this measure (see Fig. 3).

**Fig. 4:**
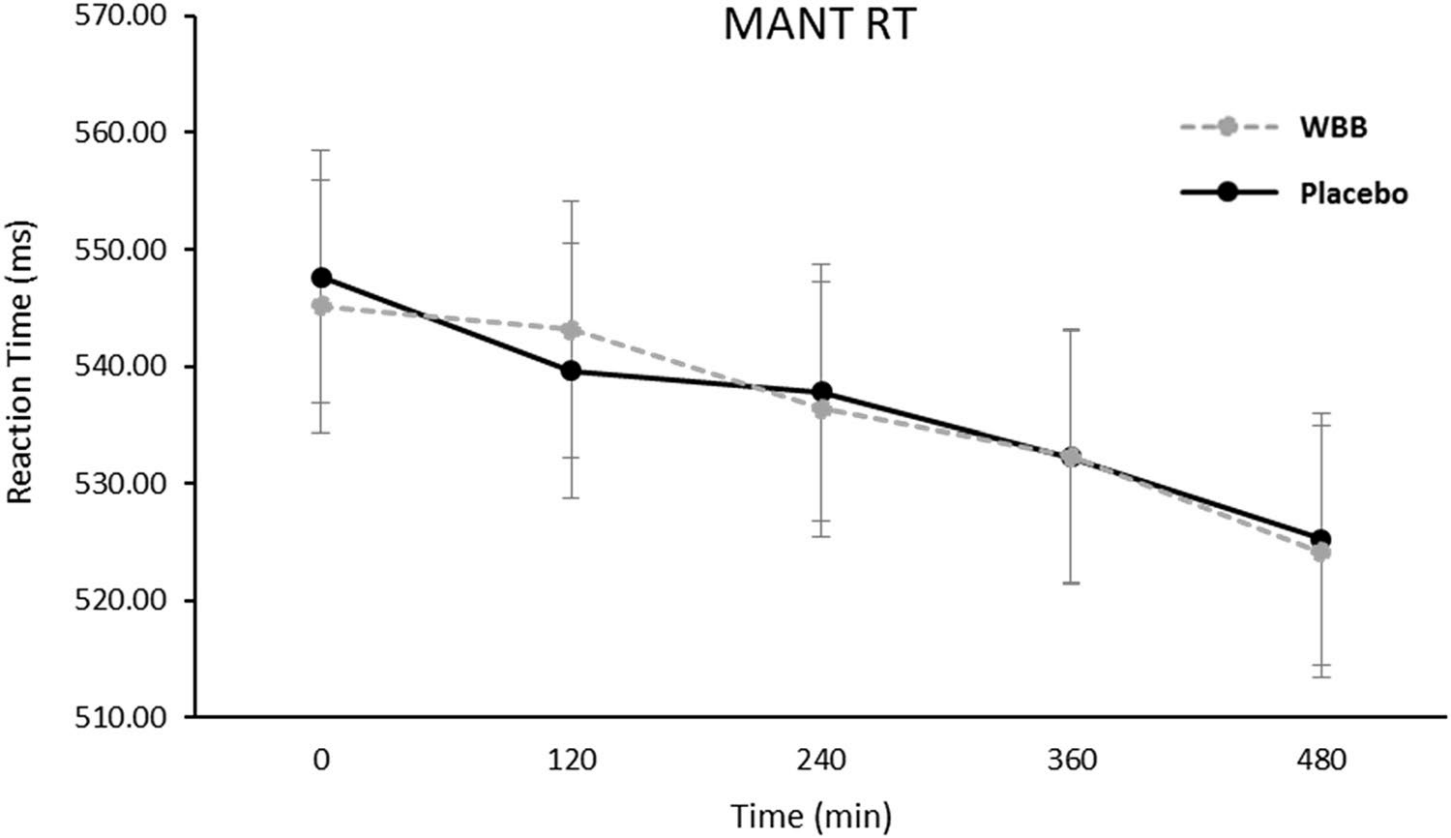
Mean reaction time (± Standard error of the mean) at all-time points following WBB or control intervention. Note, as described in the text, for both beverages, pairwise comparisons revealed significant improvements in RT between a number of time points

For the RT analysis, the expected Congruency [*F*(1,212.5) = 896.3, *p* < 0.001] and Load [*F*(1,212,5) = 76.8, *p* < 0.001] effects were observed, however, no significant treatment-related RT effects were found for this task. For both beverages, pairwise comparisons revealed significant improvements in RT between all-time points with the exception of baseline and 120 min, and 120 min and 240 min for WBB treatment, and baseline and 120 min, 120 and 240 min, 120 and 360 min, and 240 and 360 min for the placebo treatment. There were no significant between treatment pairwise comparisons for this measure (see Fig. 4).

#### Go/No-Go task

**Fig. 5:**
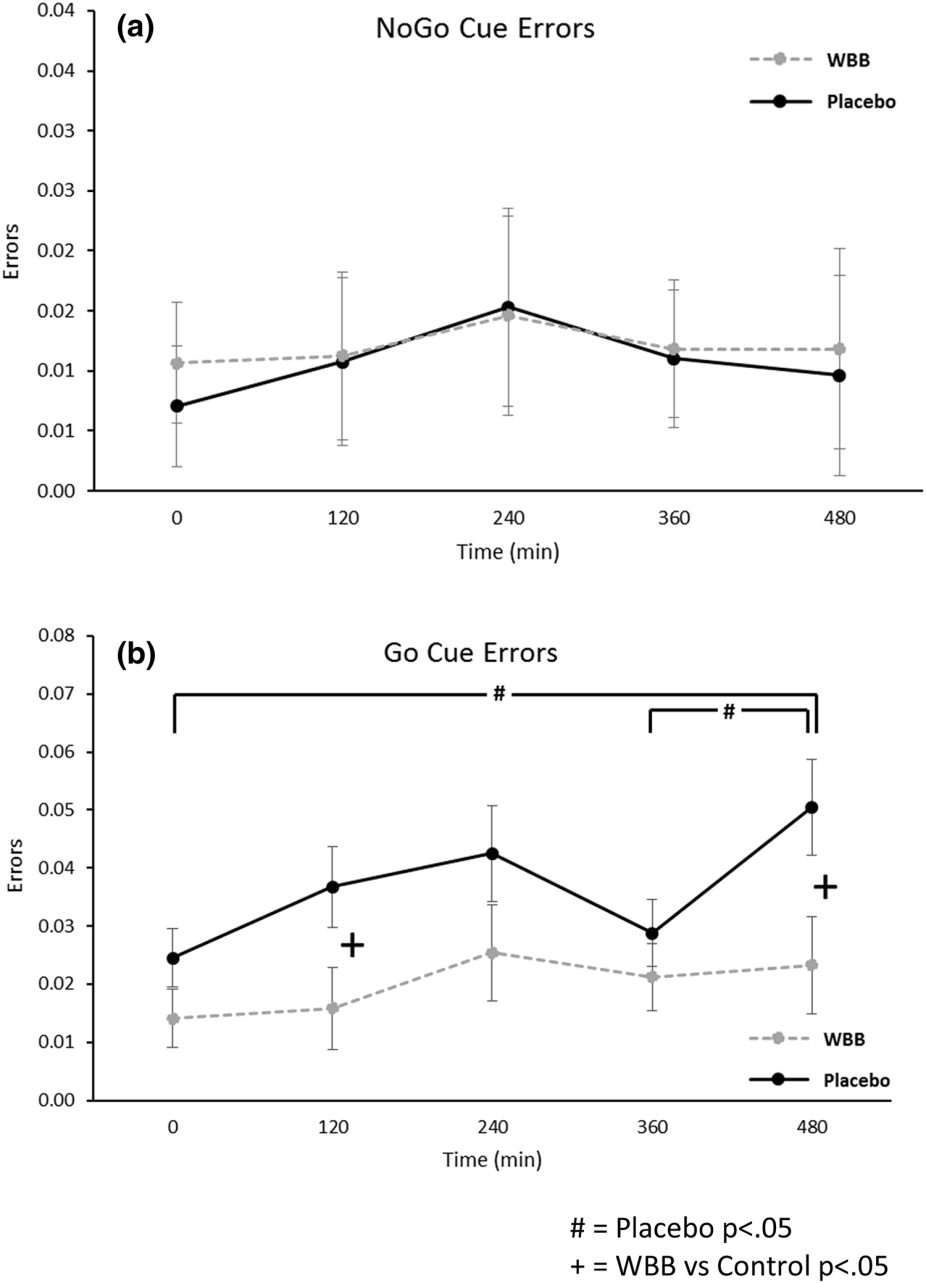
**a** Mean number of errors for congruent No-Go Cues (± Standard error of the mean) at all-time points following WBB or control intervention. **b** Mean number of errors for incongruent Go Cues (± Standard error of the mean) at all-time points following WBB or placebo intervention [significant difference between treatments, *p* = 0.018]. In comparison to WBB, there were significantly more errors for the placebo condition at 120 min and 480 min. Placebo condition errors can be seen to increase significantly between baseline and 480 min and between 360 and 480 min, whereas the number of errors remain constant for WBB

Cue type [*F*(1,104) = 11.98, *p* = 0.001] predicted errors, with ‘Go cues’ leading to more errors (*M* = 0.028) in comparison to No-Go cues’ (*M* = 0.011). A trend towards significance was found for Visit [*F*(1, 110) = 3.42, *p* = 0.067], with participants making more errors at Visit 2 (*M* = 0.024) in comparison to Visit 1 (*M* = 0.016), and for Time [*F*(4, 139) = 2.42, *p* = 0.051], with participants performing less accurately later in the day. There was no effect of Beverage [*F*(1, 104.4) = 2.47, *p* = 0.119], Beverage x Time interaction [*F*(4, 67) = 1.084, *p* = 0.371], or Beverage x Cue x Time interaction [*F*(8, 139) = 0.825, *p* = 0.582]. There was a trend towards significance for the Beverage x Cue interaction [*F*(1,104) = 3.36, *p* = 0.07]. Pairwise comparisons (via post hoc Bonferroni) revealed no beverage-related effect for the valid, less cognitively demanding, No-Go cues (see Fig. 5a). Pairwise comparisons did, however, reveal a difference between WBB (*M* = 0.02) and placebo (*M* = 0.037) for the more cognitively demanding invalid Go cues [*p* = 0.018]. Figure 5b shows the increase in errors for placebo between baseline and 480 min [*p* = 0.013] and between 360 and 480 min [*p* = 0.042] for invalid Go cues, whereas errors were maintained at the same level across the assessment points following WBB treatment. Indeed, WBB was associated with better performance at 120 min (WBB *M* = 0.016 vs placebo = 0. 037; [*p* = 0.03]) and at 480 min (WBB *M* = 0.023 vs placebo *M* = 0.05; [*p* = 0.019]).

**Fig. 6:**
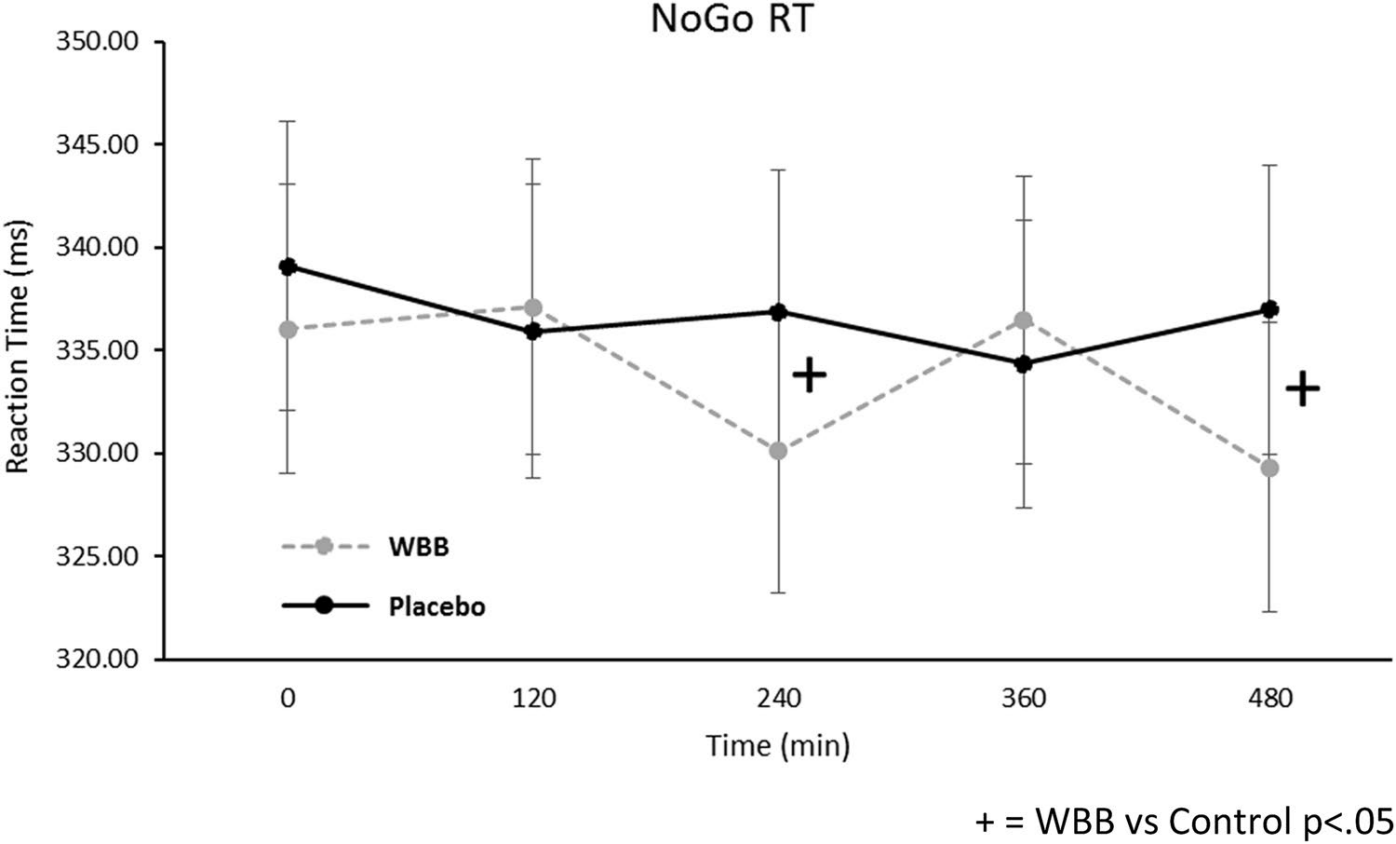
Mean RT for all Go/No-Go ‘go’ trials regardless of cue type (± Standard error of the mean) at each time point following WBB or placebo intervention. There was a marginally significant treatment x time interaction with WBB performing faster than placebo at 240 min and 480 min. Note: For the second analysis including Age, BMI, AUC_0-120_ glucose and AUC_0-120_ insulin as covariates, the main effect of treatment was marginally significant (*p* = 0.053)

Cue type also predicted RT on the Go/No-Go task [*F*(1,101) = 13.7, *p* < 0.001]. There was no main effect of Beverage [*F*(1,100.6) = 1.88, *p* = 0.173] but the Beverage x Time interaction trended towards significance [*F*(4,140) = 3.36, *p* = 0.050] and, as shown in Fig. 6, for ‘go’ trials regardless of cue type, pairwise comparisons showed faster performance following WBB in comparison to placebo at 240 min (*M* = 330 ms vs *M* = 337 ms; [*p* = 0.036]) and 480 min (*M* = 329 ms vs *M* = 337 ms; [p = 0.034]). Furthermore, at 480 min, Go trial RTs were significantly faster following WBB treatment when primed with a cognitively demanding No-Go cue (*M* = 235 vs *M* = 345; [*p* = 0.049]).

### Postprandial glucose, insulin and triglyceride responses

**Fig. 7:**
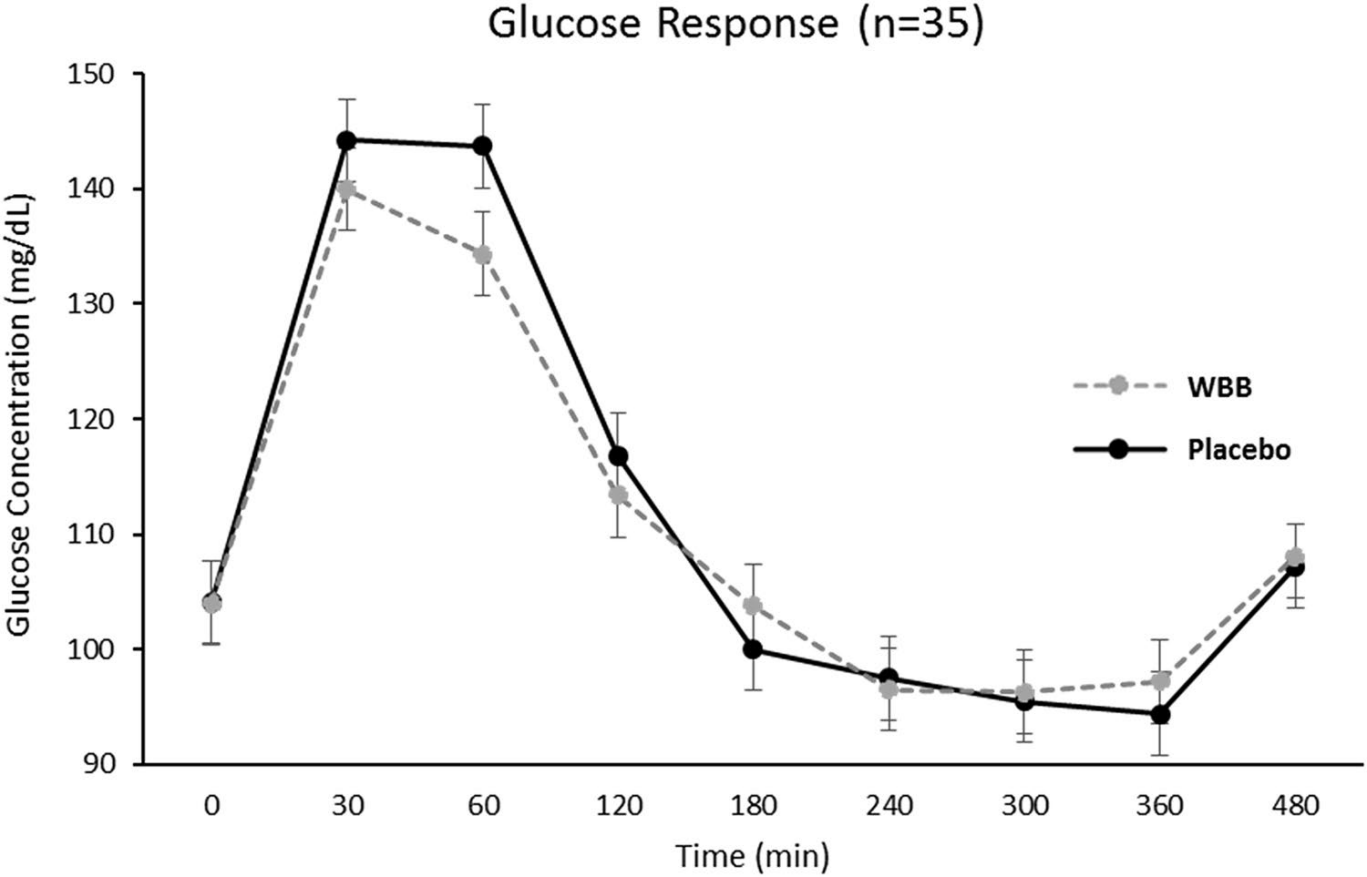
Glucose response over the 480 min following WBB and placebo treatment

**Fig. 8:**
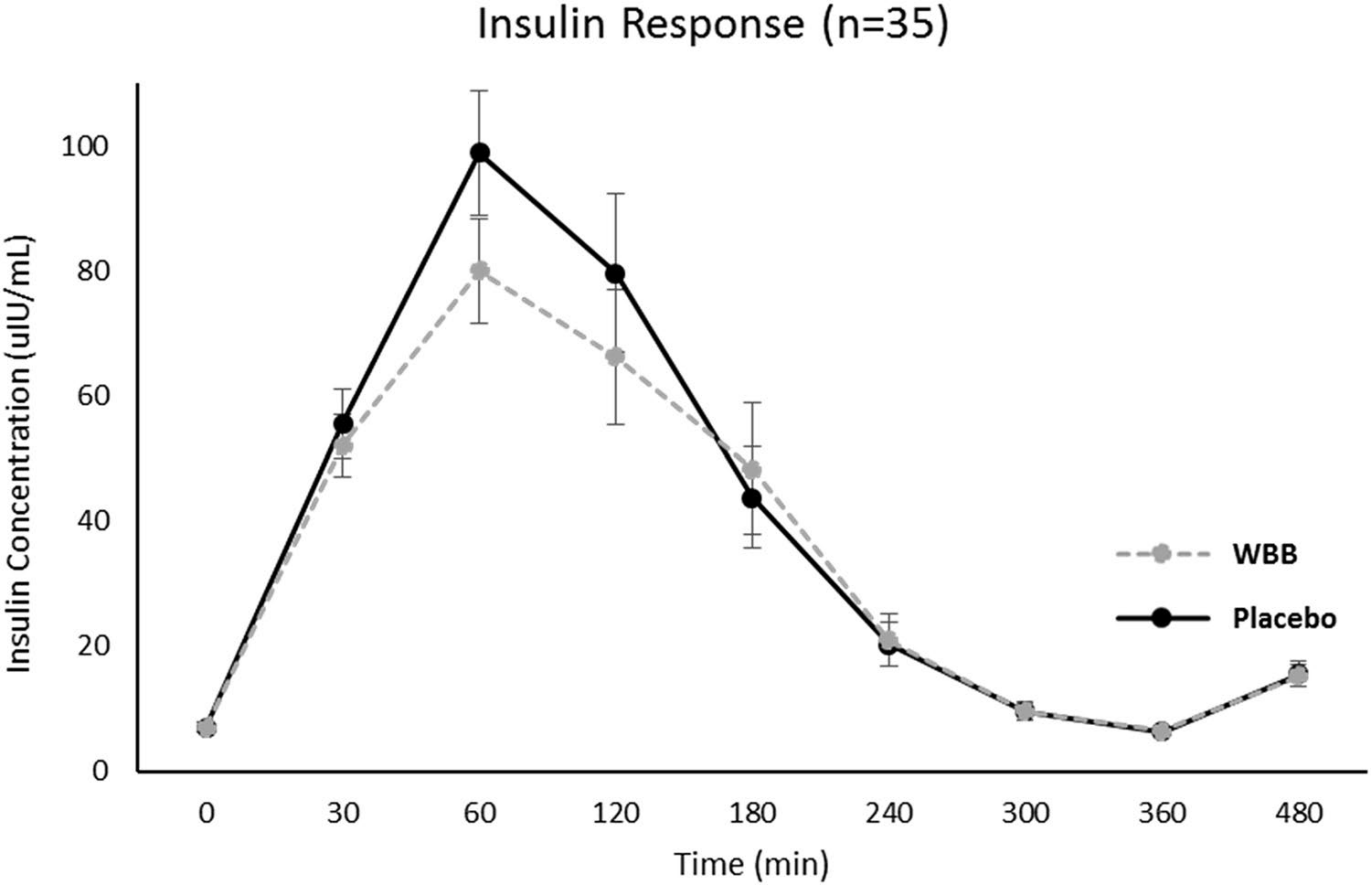
Insulin response over the 480 min following WBB and placebo treatment

**Fig. 9:**
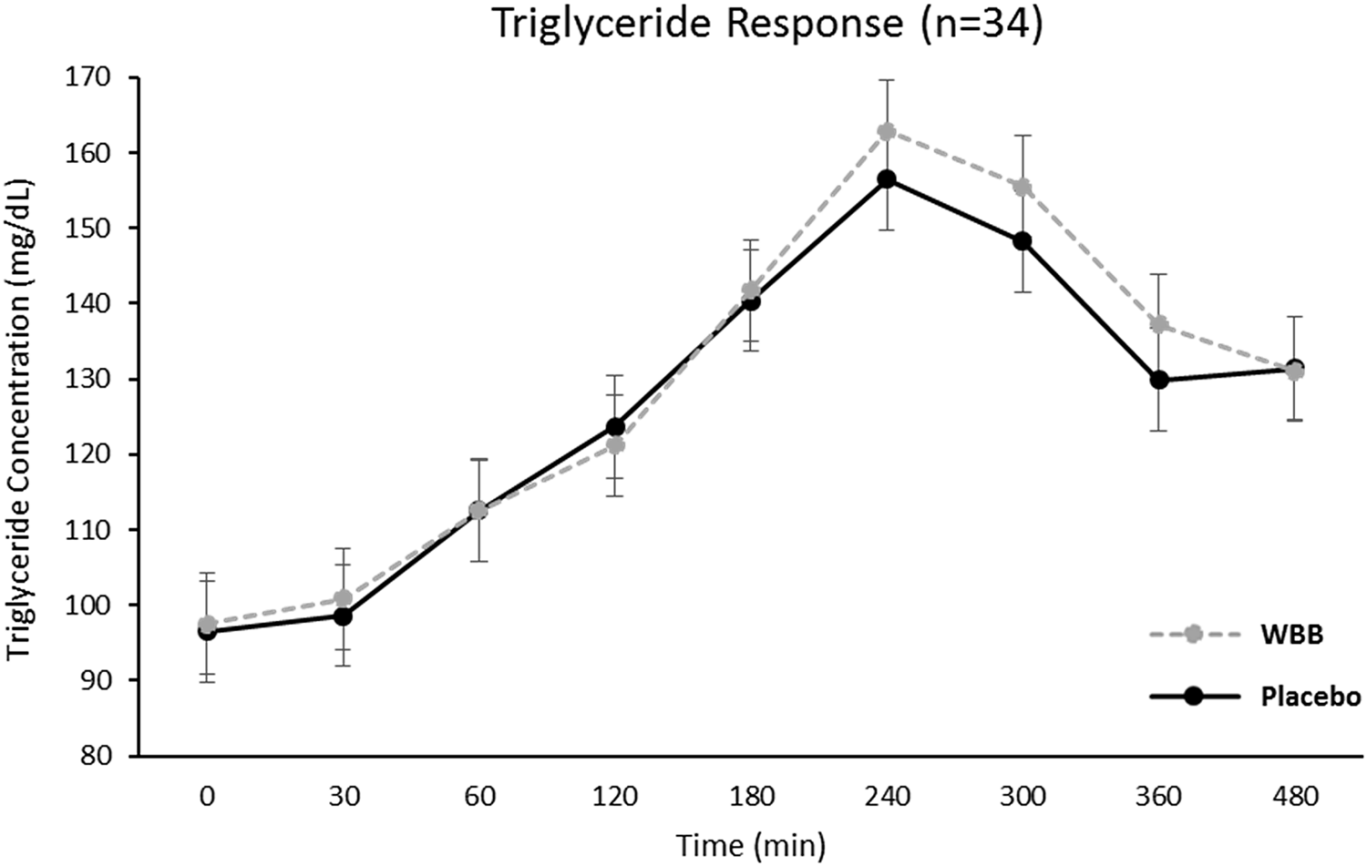
Triglyceride response over the 480 min following WBB and placebo treatment

**Fig. 10:**
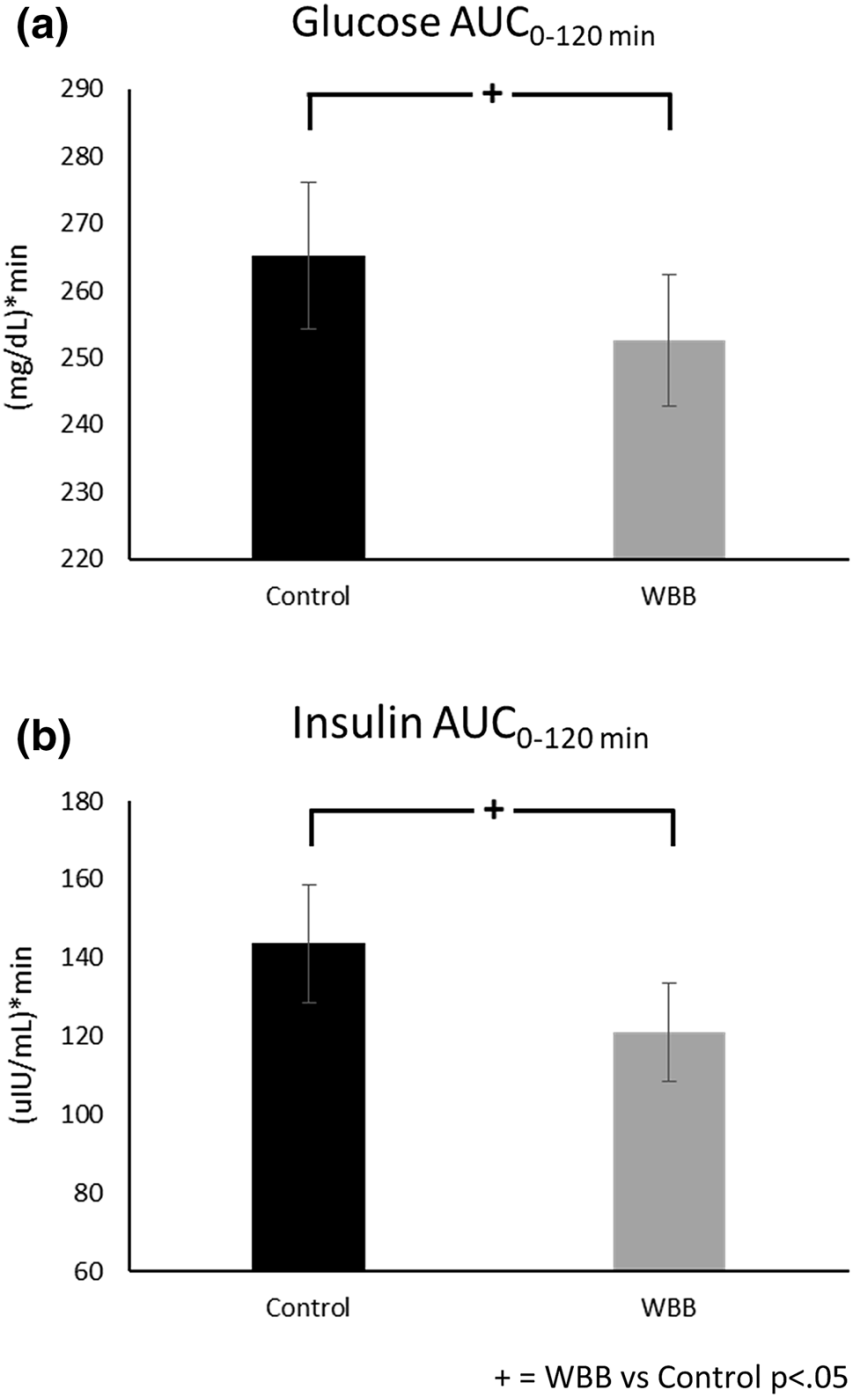
Zero to 120 min area under the curve comparisons for **a** Glucose and **b** Insulin

Postprandial glucose, insulin, and triglycerides increased after both Beverages [Time effect: glucose *F*(558) = 66.7, *p* < 0.001, insulin *F*(551) = 328.2, *p* < 0.001, triglyceride *F*(554) = 42.1, *p* < 0.001]. Peak glucose and insulin concentrations occurred at 30 min and 60 min after placebo and WBB, respectively (Figs. 7 and 8). Postprandial triglyceride concentrations peaked at 240 min after both treatments (Fig. 9). Mean postprandial glucose, insulin and triglyceride concentrations over 480 min was not different between treatments [*p* > 0.05 for all]. As shown in Fig. 10, AUC analysis of the post-meal increments of 120 min indicated a significant reduction in glucose and insulin concentrations after WBB vs placebo beverages within the first 120 min after breakfast [AUC_0-120,_ *p* = 0.03 and *p* = 0.01] but were not different between treatments thereafter [AUC_120-240_ and AUC_240-360_ and AUC_360-240_ *p* > 0.05].

### Metabolic, age, and BMI covariate analysis

#### AVLT

Including the covariates Age, BMI, and AUC_0-120_ of Glucose and Insulin in the models did not influence the pattern of significance for the episodic memory measures with the exception of Word Rejection accuracy where the main effect of Beverage was no longer significant [*F*(1,34.7) = 1.39, *p* = 0.246]. The Beverage x Time interaction trend towards significance remained [*F*(4,70) = 2.08, *p* = 0.093] as did the significant pairwise comparisons for this measure, which, in particular, suggested improved WBB performance in comparison to placebo at 240 min [*p* = 0.002].

The covariate, BMI, predicted performance or interacted with Beverage on the following measures: Immediate Recall, trend [*F*(1,38.4) = 3.89, *p* = 0.056]; Total Number of Words Learned, BMI × Beverage trend interaction [*F*(1,34.3) = 3.90, *p* = 0.056] for both WBB and placebo, as BMI increased, performance decreased [WBB beta =  – 0.139, placebo beta =  – 0.032]; Final Acquisition, trend [*F*(1,37.5) = 3.65, *p* = 0.064], there was also a BMI x Beverage interaction for this measure [*F*(1,33.4) = 4.153, *p* < 0.05] for both WBB and placebo, as BMI increased, performance decreased [WBB beta =  – 0.226, placebo beta =  – 0.147]; Delayed Recall [*F*(1,36.2) = 6.61, *p* = 0.014], there was also a BMI × Beverage interaction for this measure [*F*(1,34.4) = 4.26, *p* = 0.047], for both WBB and placebo, as BMI increased, performance decreased WBB [beta =  – 0.367, placebo beta =  – 0.244]; Word Recognition trend [*F*(1,33.2) = 3.63, *p* = 0.065].

The covariate, Age, predicted performance or interacted with Beverage on the following measures: Delayed Recall, Age x Beverage interaction [*F*(1,32.4) = 7.496, *p* = 0.010], for WBB as age increased performance increased [beta = 0.051], conversely for placebo as age increased performance decreased [beta =  – 0.020]; Word Recognition [*F*(1,30.7) = 3.22, *p* = 0.083].

The covariate AUC_0-120_ Insulin predicted performance on the following measures: Proactive Interference [*F*(1,70.2) = 7.27, *p* = 0.009]; Delayed Recall [*F*(1,66.1) = 5.03, *p* = 0.028]; Word Recognition [*F(*1,57.2) = 2.92, *p* = 0.093]. AUC_0-120_ Insulin did not interact with Beverage on any measure.

The covariate, AUC_0-120_ glucose, predicted performance on Proactive Interference [*F*(1,69.3) = 5.88, *p* = 0.018] only and did not interact with Beverage on any measure.

#### Modified attention network task

Including the covariates Age, BMI, and AUC_0-120_ of Glucose and Insulin in the models did not influence the pattern of significance for the executive function MANT measures. Furthermore, BMI and AUC_0-120_ Glucose did not significantly contribute to the models.

The Age covariate predicted performance for accuracy, trend [*F*(1,35.3) = 3.19, *p* = 0.083] and RT [*F*(1,34.8) = 9.05, *p* = 0.005]. For RT there was also an Age x Beverage interaction [*F(*1,250.8) = 8.68, *p* = 0.004], for both WBB and placebo, as Age increased RT slowed [WBB beta = 4.05, placebo beta = 3.09]. Finally, for RT, there was a AUC_0-120_ Insulin × Beverage interaction trend [*F*(1,261) = 3.667, *p* = 0 0.057] for WBB as AUC_0-120_ Insulin increased RT became faster [beta =  – 0.067], conversely for placebo as AUC_0-120_ Insulin increased RT slowed [beta = 0.018].

#### Go/No-Go

For the Go/No-Go RT analysis, when including the covariates age, BMI, and AUC_0-120_ of Glucose and Insulin the main effect of cue type remained a significant predictor of RT [*F*(1,102.6) = 15.8, *p* < 0.001]. Importantly, Beverage was found to marginally predict RT [*F*(1,105.7) = 3.82, *p* = 0.053], RT was faster after the WBB Beverage compared to placebo (*M* = 333 ms vs *M* = 338 ms, respectively). There was also a significant Beverage × Time interaction [*F*(4,139.9) = 2.44, *p* < 0.05] with pairwise comparisons revealing the same pattern of results as seen previously. The AUC_0-120_ Glucose and BMI covariates did not significantly contribute to the model. The Age covariate predicted RT [*F*(1,35.7) = 13.48, *p* = 0.001]. Furthermore there was an Age x Beverage trend [*F*(1,108.5) = 3.32, *p* = 0.071], for both WBB and placebo as Age increased RT also slowed [WBB beta = 2.80, placebo beta = 2.33]. The AUC_0-120_ Insulin covariate predicted RT [*F*(1,140.3) = 9.96, *p* = 0.002], but did not interact with Beverage.

Including the covariates age, BMI, and AUC_0-120_ of Glucose and Insulin in the model did not influence the pattern of significance for the Go/No-Go error measures. The AUC_0-120_ Glucose and age covariates did not significantly contribute to the model. The covariate AUC_0-120_ Insulin predicted number of errors [*F*(1,44.6) = 4.24, *p* = 0.045], but did not interact with Beverage. There was a BMI x Beverage interaction trend [*F*(1,108.5) = 2.89, *p* = 0.092] whereby for WBB as BMI increased there was a small increase in number of errors [beta = 0.002] and for placebo, as BMI increased there was a small decrease in number of errors [beta <  – 0.001].

## Discussion

This study considered for the first time the relationship between cognitive behaviour and metabolic responses following a single polyphenol-rich WBB drink with a meal in middle-aged adults. Similar to previous research with other age groups, WBB cognitive performance was improved in comparison to placebo on delayed recognition memory and aspects of executive function [16–21]. Importantly, these benefits were found on more demanding elements of the tasks where some form of additional cognitive effort was required. For the AVLT, rejecting foil words in the word recognition component of the AVLT would require the participant to overcome interference from the previously presented list B items and 20 novel foils as opposed to recognising the list A items which had received repeated encoding over a number of trials. For the Go/No-Go task, inhibiting No-Go responses following a misleading ‘Go’ cue, and responding faster following a misleading No-Go cue requires additional cognitive effort to overcome the pre-potent effect of these cues which is not present where a facilitating congruent cue is given. Furthermore, it is interesting that particular differences were found between treatments at 120 min for the ‘Go’ cue errors, coinciding with previous reports of elevated WBB metabolites in the blood [25] and increased flow mediated vasodilation [40]. Differences were also found at 480 min again for ‘Go’ cue errors and also for invalid ‘NoGo' cue RT, the final test session of the day, presumably the point at which the participants had reduced cognitively resources. Although it should be noted that further differences were found at 240 min for the Word Rejection measure, and general Go/No-Go RT, indicating some evidence of WBB benefit in the absence of cognitive fatigue. Taken as a whole, these cognitive findings are in line with previous WBB research in children [19] and indicate that consuming a whole fruit WBB drink with a meal, compared to an energy-matched non-WBB drink, enhanced performance on higher demand cognitive tasks in middle-aged participants.

Whilst flavonoid-rich blueberry interventions have demonstrated improvements in measures of memory and executive function following chronic intervention [6, 10–12], Bell et al. [4] have noted that different cognitive domains appear affected following acute flavonoid/polyphenol interventions dependent on the age of the participants tested with memory improvements generally being observed in children and older adults, executive function improvements in children, younger, and middle-aged adults, and working memory improvements more typically observed in younger and middle-aged adults. Findings from recent acute flavonoid-rich berry interventions have been broadly consistent with this observation. For young adults, improved executive function performance has been demonstrated following acute grape juice [41], and mixed berry [21] intervention, and improved working memory has been shown following a grape/blueberry extract [42]. Conversely, for older adults, improved episodic memory word recognition and word recall has been found following haskap berry [43] and a recent blueberry intervention study found improvements on a global measure of cognitive performance which, when the individual tasks were considered separately, revealed improved word recognition and also a trend for improved performance on an EF switching task [22]. In the current study, improvements were found in both episodic memory and executive function performance. Although the memory effect involving the rejection of interfering words during the word recognition task might also be considered a consequence of executive control, it is interesting to note that the memory benefits found here are in line with the recent studies described above which also showed berry-related benefits on word recognition measures. The executive function benefits were primarily observed in the response inhibition Go/No-Go task, with little evidence being shown for a treatment effect on the MANT. Interestingly, in an fMRI study, Blasi et al. [44] found greater pre-frontal cortex activation during response inhibition tasks in comparison to response interference tasks, which showed greater nucleus accumbens (ACC) activation. Research by Dodd [45] has indicated that blueberry intervention increases cerebral blood flow to pre-frontal areas but not the ACC. It is therefore plausible that there is a selective increase in blood flow to brain areas mediating response inhibition performance on the Go/No-Go task but not response interference performance as was observed in this study.

Metabolic responses to treatments were different during the first 120 min post-meal (i.e., AUC_0-120_) with significantly lower glucose and insulin recorded for WBB in comparison to placebo. The addition of these variables to the model as covariates, along with BMI and Age, had varying impact depending on the cognitive variable investigated. The main effect of treatment for word rejection was no longer significant; however, the covariates did not significantly contribute to the model or interact with treatment. Furthermore, the interaction trend and significant pairwise comparisons remained consistent for the word rejection measure.

For the Go/No-Go task, however, the addition of the four covariates to the model introduced a marginal trend effect for faster RT performance for the Go/No-Go task in conjunction with WBB beverage consumption. Though both AUC_0-120_ insulin and age contributed significantly to the model for Go/No-Go RT, only Age interacted with Beverage as a trend and it therefore seems probable that age is the factor most likely to be contributing to this RT effect. As noted above, cognitive abilities, especially memory and executive abilities, decline with age and the interaction here between Age and Beverage provides further evidence for the efficacy of WBB treatment in ameliorating age-related cognitive decline. For a number of other measures, age either predicted performance or interacted with treatment, but did not significantly change the previously observed pattern of results.

BMI predicted performance and interacted with treatment on several AVLT measures and also interacted as a trend with treatment on the Go/No-Go errors measure. As might be expected from previous research [46], the relationship between BMI and cognitive performance was uniformly negative; the only exception being the weak Go/No-Go interaction trend. Where the covariate analysis revealed a significant change in the main effect of treatment on Word Rejection and Go/No-Go RT, BMI did not contribute significantly to the model. Therefore, from the results of this particular study, it might be concluded that whilst the significant interactions give some evidence of a relationship between BMI and treatment, this is not sufficiently strong to impact the effect of treatment in this middle-aged sample.

It is interesting to note that AUC_0-120_ Glucose only contributed significantly to the model for proactive interference and did not interact significantly with treatment on any measure. However, AUC_0-120_ insulin contributed significantly to three AVLT variables and the Go/No-Go Errors and RT measures, and interacted with treatment (as a non-significant trend only) for MANT RT. Whilst this demonstrates an apparently stronger relationship between insulin and cognitive function than between glucose and cognition, this would seem to be primarily independent from treatment in the middle-aged sample considered here.

Our metabolic findings are consistent with recent younger adult findings, which have shown postprandial glucose concentrations to be attenuated following a similar 517 mg anthocyanin dose to the estimated 475 mg dose used in the current study [47, 48]. Furthermore, in the same study, improved cognitive function was demonstrated on the demanding serial 7 s task at one and two hours following intervention (cognitive testing did not take place beyond two hours) [47]. Whilst, this converging evidence provides strong support for both cognitive and metabolic benefits following WBB intervention, the lack of a statistically significant interaction between treatment-related changes in glucose or insulin concentrations and cognitive performance indicates that the underlying mechanisms may be more complex than blood glucose control alone. Another consideration would be that this study did not possess adequate statistical power to detect these associations, especially in the context of a strong effect for Age. Our overall power was approximately 70%, which is a general limitation for this study. However, it is sufficient to provide insights supporting further consideration of the way in which metabolic and cognitive factors interact with other physiological responses. For example, the three-way interaction between glucose availability and or insulin sensitivity, increased blood flow, and cognitive performance may prove a fruitful avenue for further research.

The research had many strengths and limitations. Though previous research has considered younger adults, this is the first research to examine WBB effects relative to metabolic responses in a middle-age population acutely. While other studies examine effects of treatments in a well-rested state, typically after chronic intervention, our paradigm tested acute effects in a relatively fresh-to-fatigued model over the course of a day. The model also induced a metabolic challenge by providing subjects with a typical Western style meal to drink with treatment beverages. Limitations included a small sample size, although thirty-five subjects is larger than most berry trials and the cross-over design increased power as subjects served as their own controls. Assessment of WBB metabolites would have allowed for an extra variable to consider in the model, as individual variability in flavonoid metabolism has been documented, which may have helped explain results.

## Conclusion

Here we have presented the effects of a polyphenol-rich, wild blueberry intervention on acute cognitive function and metabolic outcomes in middle-aged adults. The findings provide further support for the efficacy of wild blueberry on improving cognitive outcomes within this age group, particularly where there is increased cognitive demand. Wild blueberry was also found to reduce glucose and insulin concentrations in response to a meal over the initial 120 min having implications for post-meal metabolic control. Although there was little evidence of a direct relationship on cognition, these data have importance for structuring meal plans to reduce the metabolic burden in individuals with glucose homeostasis concerns.
